# Understanding international perceptions of the severity of harmful content online

**DOI:** 10.1371/journal.pone.0256762

**Published:** 2021-08-27

**Authors:** Jialun Aaron Jiang, Morgan Klaus Scheuerman, Casey Fiesler, Jed R. Brubaker

**Affiliations:** Department of Information Science, University of Colorado Boulder, Boulder, Colorado, United States of America; University of Oxford, UNITED KINGDOM

## Abstract

Online social media platforms constantly struggle with harmful content such as misinformation and violence, but how to effectively moderate and prioritize such content for billions of global users with different backgrounds and values presents a challenge. Through an international survey with 1,696 internet users across 8 different countries across the world, this empirical study examines how international users perceive harmful content online and the similarities and differences in their perceptions. We found that across countries, the perceived severity consistently followed an exponential growth as the harmful content became more severe, but *what* harmful content were perceived as more or less severe varied significantly. Our results challenge platform content moderation’s status quo of using a one-size-fits-all approach to govern international users, and provide guidance on how platforms may wish to prioritize and customize their moderation of harmful content.

## Introduction

It is no secret that social media platforms have been plagued with harmful content—issues like misinformation and the incitement of violence. As a result, platforms actively moderate content to prevent users from harm and free their sites from abuse. However, as social media platforms have grown to a global scale, the people, and their associated values and behaviors, that platforms need to moderate have also become increasingly diverse [1]. As a result, moderating the enormous amount of content they generate becomes a significant challenge. Take Facebook, currently one of the largest social media platforms, which has 2.7 billion monthly active users worldwide and supports 111 languages. Despite its size, it has about 15,000 moderators [2] compared to billions of its users. It also has one set of rules, the Facebook Community Standards [3], guiding that moderation.

Facebook moderators need to enforce the Community Standards consistently, throughout the world—but how do we ensure consistency when the moderators and the people they moderate come from different backgrounds and cultures? For example, prior research has shown that the acceptability of sexualized nudity varies between cultures [4], and exiting news reports have also shown that translation of community guidelines did not extend to all languages [2]. When a moderator in Hyderabad needs to review content created by someone in Austin, as a result of globally distributed moderation [5], do they perceive the content and the governing rules consistently? Furthermore, as the moderation resources are limited compared to the extremely large amount of content that they need to handle, how should platforms prioritize moderation resources? Should that prioritization change in different parts of the world?

Prior research (e.g., [6, 7]) has suggested that *severity* can be a good heuristic to differentiate and prioritize different types of harmful content; violations that are more severe should be prioritized than those less severe. Severity, in this case, refers to the intensity of harm of violating content. From a severity prioritization perspective, a deep understanding of severity is necessary for actionable guidelines for moderators to make more informed moderation decisions, for social media platforms to more effectively prioritize moderation capacity, and for regular users to better understand online rules. A severity prioritization perspective can also guide the different levels of consequences of violating the rules.

Therefore, in this paper, we quantitatively investigate the severity of different types of harmful content through a survey with people from eight different countries in the world. In presenting our work, we first introduce prior work on social media content moderation. We then detail how we developed our survey, highlighting a novel method to measure participants’ perceptions of the severity of identified types of harm: willingness to fine. We present each method of analysis alongside findings by research question. In presenting the findings, we answer the following research questions:

- How do people in different countries perceive the severity of harmful content?
- What are the similarities and differences between perceptions of severity in different countries?
- How do countries agree or disagree on the severity of different types of harmful content?
- What are some sensitive topics for specific countries?

## Background: Content moderation on social media platforms

Social media platforms, like Facebook, Twitter, and YouTube, have become a central part of the social lives of billions of people across the world. While these platforms are not by themselves content producers, they are responsible for storing, organizing, and circulating a massive amount of user-generated content [5, 8]. Despite their claims of being impartial and their reluctance to regulate speech [1], many platforms are incentivized to moderate. They must not only meet legal and policy requirements, but also avoid losing users subject to malicious behaviors, protect their corporate image, placate advertisers who do not want to be associated with negative online communities, and honor their own institutional ethics [8, 9]. Through a case study of Reddit’s ban on hate communities, prior research has shown that these moderation efforts can be effective in improving otherwise toxic online communities [10].

Platforms often govern users with two types of rules: terms of service and community guidelines. Terms of service serve a legal contract between the platform and the users; they spell out each party’s obligations and liabilities [1]. Jackson et al. have called for deeply integrating the role of policy into research, pointing out that policy is deeply intertwined with design and practice [11].

Community guidelines, on the other hand, often use plain language that explains platforms’ expectations of proper user behavior. Compared to terms of service, users are more likely to read community guidelines and understand them when they have the need to reference platform rules [1]. Platforms often impose community guidelines that detail acceptable and unacceptable behaviors. They prohibit illegal behaviors, such as engaging in child exploitation and human trafficking, but they also police non-illegal upsetting content, like harassment and commercial spam that could drive users away. Gillespie argues that these rules are important for numerous reasons. On one hand, they contain abusive behaviors and set the norms of platforms. On the other, they also help construct an ecosystem of governance that smaller platforms may look to for guidance, sometimes borrowing their languages directly [12]. Larger platforms may also adjust rules and policies in relation to each other [8]. A recent example is Twitter’s ban on political ads immediately following Facebook’s reluctance of removing or fact-checking them [13]. These rules, especially when they are different, reveal how platforms see themselves as the arbiters of cultural values.

As platforms grow to have millions, even billions of users who produce massive amounts of content, scale presents significant challenges to platform content moderation [5]. The challenge of scale is two-fold. First, the vast amount of content eliminates the possibility of *ex-ante*, or proactive moderation, where moderators review content before it can appear on the platform. However, almost all platforms have to resort to *ex-post* moderation, or reactive content moderation, which initially allows all content to be published without review. Moderators then remove or filter questionable content after the fact, often after it has been reported by users [5, 8]. However, this approach results in malicious content impacting users on the platform, even if it only stays up for a short period of time. Second, moderating an immense amount of content also requires a large human workforce. In 2019, Facebook alone had 15,000 full-time moderators across the world to combat malicious content, but this number is clearly outpaced by the billions of generated content they must review [14]. Some disturbing content therefore remains online for days, months, or even years due to the lack of moderation capacity among other complications in large scale content moderation [1]. Many malicious users also try to circumvent moderation such as by using lexical variations, which makes timely moderation even harder [15, 16]. Platforms have been using automated algorithms to assist with removing abusive content [17], but the accuracy of algorithmic moderation still has much to be desired.

As platforms become global, platform rules start to intersect with the various but granular local laws [18]. For example, the EU has rules against certain types of hate speech [19]. Such is also the case with holocaust denial. While many platforms commit to remove such content, it is only a legal requirement in certain countries including Austria and Germany [20]. At the same time, the massive amount of users from various cultures and backgrounds also bring a wide variety of norms and values, presenting another challenge to scaling up human moderation labor [1], especially for platforms with a single set of rules for users across the world. Popular news has been rife with controversial moderation decisions. For example, when Facebook moderators removed the Pulitzer Prize-winning Vietnam war photo featuring children fleeing a napalm attack for child nudity [21], or when Facebook and Twitter seemed to exempt politicians spreading misinformation from removal or fact-checking. The debates about how to handle moderating contentious topics, like prize winning photos and misinformation, reflect a clash of different backgrounds, purposes, and values. Recent work has argued for a “constitutional layer” in digital institutions to make changes that are sensitive to local contexts [22]. Our research speaks to potentially highlighting this clash of values by examining how people from different cultures and countries perceive rule violations.

Regardless of a proactive or reactive approach, human moderators need to review significant amount of content, whether violating or not, 24/7. To meet such high moderation demand, many platforms resorted to outsource content moderators [23]. In her work about commercial content moderators [5], Roberts pointed out that the factory-like routine of content moderation work has led many moderators to burn out. The constant viewing of disturbing and traumatizing content takes a heavy emotional toll on the moderators, who are reluctant to discuss their work with their family and friends to avoid burdening them [14].

## Methods

In this study, we deployed a survey globally to capture regular internet users’ perceptions of the severity of harmful online content. In this section, we first detail how we implemented the survey, including survey question development, participant recruitment, and language translation. We then describe our data cleaning procedures in preparation for our analysis. The full survey instrument as well as participant response data are included in S2 File.

### Survey development

Given that the goal of our study is to uncover culturally situated perspectives of harmful content online across the globe, we designed the survey with two primary goals: to be both (1) generalizable across platforms, and (2) representing a variety of countries.

#### Survey questions

To ensure the generalizability of survey results to different platform community standards, we asked the participants to rate each type of harmful content identified in Jiang et al.’s cross-platform content analysis [24]. Because participants may not have the same understanding about what each type of harmful content was, we asked participants to consider and rate each type of harmful content within the context of a hypothetical scenario, where they needed to imagine someone violated a particular rule. For example, participants rated the severity of “revenge porn” by responding to a hypothetical scenario where they see someone posted photos of revenge porn. We also described the scenarios as if they happened on Facebook, specifically, for two reasons. First, Jiang et al.’s analysis showed that Facebook’s Community Standards was the only one that covered every type of harmful content identified. Second, concretely grounding the scenarios to a single platform could also mitigate the potential platform-related variability in perceptions of the same harmful content (e.g., people may perceive harassment differently on Facebook than on Twitter).

We employed a novel method for assessing the perceived severity of harmful content, given the limitations of common survey measurement instruments. The major limitation we needed to address when designing our survey was that inherently bounding the concept of “severity” could cognitively bias participant responses. Further, it would inherently bound severity to a linear scale—rather than a potentially exponential one. While Likert scales are a type of ordinal scale are common for quantifying respondents’ opinions in survey design, using a bounded scale that typically has only five or seven options would also limit our ability to capture the nuanced perceptions between 66 different types of harmful content, and to model an accurate relationship of severity. Therefore, we asked participants to directly report how they perceived the severity of harmful content through free-text numerical values, following an established alternative to Likert scales in psychology research [25, 26]. In other words, participants could freely input a number as their answer. A free-text input would both: (1) not limit participants to a bounded linear scale in their assessments of severity; and (2) allow us to examine the rate at which severity increased (e.g., linearly or exponentially).

As the severity of harmful content can be an highly contextual and vague concept to regular users, in the survey we operationalized severity with two more concrete concepts: punishment and urgency. The construct of *punishment* represents the negative consequences a user believe is deserved for a certain type of harmful content. Degrees of punishment is rooted in the concept of proportionality in criminal justice literature: the more severe the harm is, the graver the associated punishment should be [27]. The construct of *urgency* addresses platforms’ problem of prioritization, which in practice guides the operation of many emergency response systems [28]: the more severe the harm is, the sooner it should be taken care of.

To measure the two constructs of punishment and urgency, inspired by the willingness-to-pay measurement in economics research [29], we asked participants to respond with the amount of money that: (a) they would fine the person for posting the harmful content; and (b) the social media company should spend to remove the harmful content immediately, over all other types of harmful content. In answering these two questions, participants could input any number as low as zero (which indicated that they believed the content was not harmful), and as high as they would like. To compare the results between the free-text numeric measurements and traditional Likert-scale measurements, we also asked participants to rate the severity on a Likert scale that measured how upset the participant would be upon seeing the harmful content.

As such, participants answered the same three questions for all scenarios that they saw. Below, we show an example of both the scenario and accompanying survey questions a participant would see (in this specific example, we ask about “hate speech: dehumanization”):

Imagine you saw:

A post that says **people from a certain country are insects** on Facebook.

- How much money, if any, would you fine the person who posted this content? Please indicate your answer in {local currency}. You only need to enter a number. (Punishment measurement, free-text numeric)
- How much money, if any, do you think Facebook should spend to remove this content immediately over other types of content? Please indicate your answer in local currency. You only need to enter a number. (Urgency measurement, free-text numeric)
- How upsetting is this content to you, if at all? (Upsettingness measurement, Likert scale: extremely, very, somewhat, a little, not at all upsetting)

#### Recruitment

For the survey results to be representative of multiple countries, we strategically recruited survey participants from eight different countries through Qualtrics panels, based on published statistics of countries with the most Facebook users [30]. While this is certainly not a comprehensive list of countries for the overall social media landscape, we feel that this sample has the level of diversity needed for the purpose of our research. We recruited 1,696 participants in total, with approximately 200 participants per country. This sample size was the result of budget constraints, having approximately equal representation per country, and having a sample size with a reasonable margin of error. For example, India, which had the largest Facebook user base, had 269 million Facebook users as of October 2019. Therefore, a sample size of 200 could give a satisfactory 7% margin of error with a 95% confidence level.

The participation criteria were that the participant was over 18 years old and had used Facebook in the last 30 days. To get the most accurate sample of Facebook users, we did not specify that we were specifically looking for Facebook users. We asked participants to choose all the social media platforms that they used from a list of options, but they would only be qualified if they had chosen Facebook as an option. This sampling process was necessary as the survey scenarios were described in the context of Facebook. Following recommendations in survey methodology literature [31], we also had participants answer a commitment question in the beginning that asked whether they agreed to thoughtfully provide their best answers, and would be screened out if they could not promise to do so. We also collected demographic data including age range, gender, and education level at the end of the survey to prevent early drop-off.

#### Piloting and translation

We piloted the survey with 36 pilot participants to test the validity of our approach. All pilot participants were able to understand the questions correctly and provide reasonable answers. However, according to their feedback, asking about all 66 types of harm would make the survey overly lengthy, with a completion time of approximately 30 minutes. Therefore, to prevent fatigue and drop-off, in the final version of the survey, each participant saw a random 33 types, half of the 66 total types of harmful content. The randomization effectively made each type of harm receive approximately 100 ratings per country. While we acknowledge that the sample size could only provide limited statistical power to generalize to the population, we feel that the diversity of the respondents could still reveal useful insights.

Finally, since many parts of the world do not speak English as the dominant language, we also had the survey professionally translated into the corresponding dominant language spoken in each country. The {local currency} placeholder was also translated into the country’s dominant currency. For example, U.S. participants would see “U.S. Dollars (USD),” while Turkish participants would see “Türk Lirası (TRY)” (“Turkish Lira” in the Turkish language). Table 1 shows the final list of countries where we deployed the survey, as well as the languages into which the survey was translated. The University of Colorado Boulder Institutional Review Board approved this study (Study Number 19–0695). Consent was given digitally, via online consent forms.

**Table 1:** Recruitment and translation details for each country in the study.

Country	Language	# Participants
Brazil	Portuguese	211
Egypt	Arabic	207
India	Hindi	213
Indonesia	Indonesian	217
The Philippines	Filipino	220
Turkey	Turkish	204
United States	English	215
Vietnam	Vietnamese	209

### Data cleaning

First, we had Qualtrics perform basic data quality checks to filter out obviously poor responses or non-responses. The checks included filtering out duplicate respondents, response time that was unreasonably long or short, and patterned responses, such as all zeros (“straight-liners”). We also conducted device IP address lookups to ensure the respondent was physically in the country we were intending to gather data for. we then conducted our own data cleaning, which involved two steps:

- **Winsorization**: Because some participants entered spuriously high values for highly severe harmful content (e.g., a $10^17^ fine), we winsorized the free-numeric responses at 95% level (i.e., capping all values in the top 5% at the value at the top 5% point) to reduce the effect of extreme outlier values.
- **Normalization**: Because the absolute monetary values varied depending on each participants’ own conception of money, as well as the currency being used, these raw values were not comparable across different people or across countries. Therefore, using min-max scaling, a common normalization technique that scales raw values within a certain range, we normalized the monetary values to a maximum of 550,000, which is the median of the maximum values that the participants entered. Specifically, we used the following normalization formula:
  $$\begin{array}{c} x_{normalized}=\frac{(x-min(x))\times 550000}{max(x)-min(x)} \end{array}$$ (1)
  where **x** was each participant’s response to the 33 types of harm that they were presented, represented as a vector.
  The normalization scaled all participants’ response values to the same range of values, and therefore made them comparable with each other for our analysis. It also preserved the relative relationships between the numerical ratings for each participant.

## How do people in different countries perceive the severity of harmful content?

We first explored how participants in each country viewed harmful content broadly, and how these perspectives compared with each other when juxtaposed. Because one of the goals of the study was to understand how different types of harmful content should be prioritized, we examined how each country prioritized harmful content, reflected in their rankings. Given that each country also quantitatively rated the severity of each type of harm, we also investigated the rate of growth of severity values, as a rank order is inherently linear and may not reflect the true relationships between category types.

To understand how participants in different countries perceive the severity of content types, we first calculated the mean punishment and urgency values for each type of harmful content across participants, for each country. We then calculated the overall severity value of each type of harm as the mean of its punishment and urgency values, for each country. In other words, if *s*_*k*_ is the severity value for harmful content *k*, then

$$\begin{array}{c} s_{k}=\frac{\left(\bar{P_{k}}+\bar{U_{k}}\right)}{2} \end{array}$$ (2)

where $\bar{P_{k}}$ is the mean punishment values for *k* across participants, and $\bar{U_{k}}$ is the mean urgency values for *k* across participants. We then plotted the overall severity values of each type of harmful content against its rank order by country.

**Fig 1:**
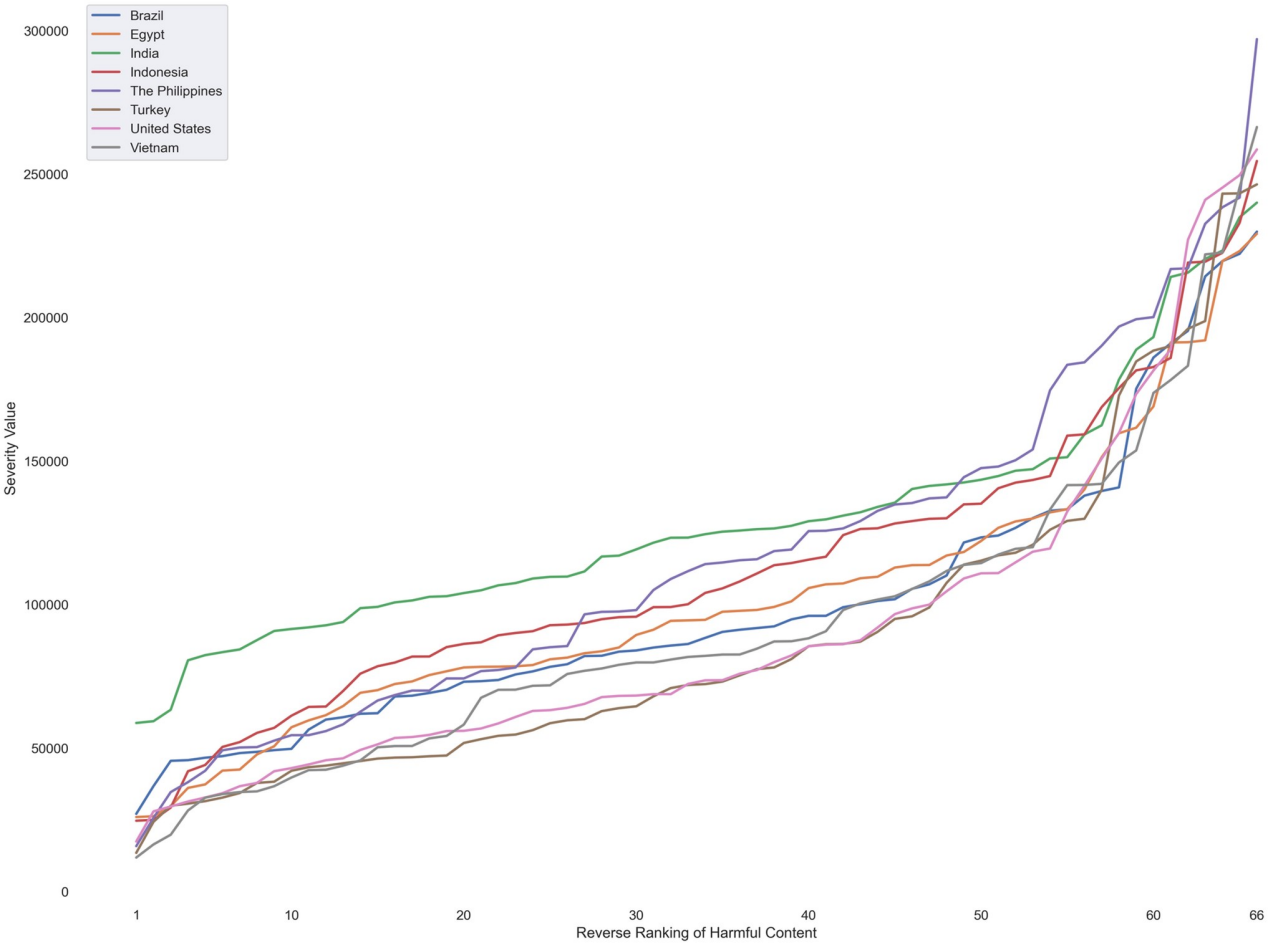
Plot of severity value vs. reverse severity rank order for each country under a free-text numeric measurement. Note that the same rank position may indicate different harmful content for different countries.

As shown in Fig 1, we observed exponential growths in the severity of harmful content consistently across countries. To confirm the exponential growth, we conducted an exponential regression on each country’s data using the statsmodels Python package. Note that we conducted regressions separately for each country, so the data used per regression was not nested in structure. Our randomization mechanism also precluded us from using a linear mixed model, as individually ranked harmful content are not likely to retain their rank positions on the full range of harmful content (i.e., hate speech with a rank position of 16 among 33 harmful content types is not likely to stay ranked the 16th when considered among 66 harmful content types).Table 2 shows the regression results using the exponential function *y* = *Ae*^*kx*^, where *y* is the severity value for each type of harmful content, and *x* is the reverse rank order of each type of harmful content (i.e., 1 = lowest, 66 = highest, in the same order as the *x*-axis in Fig 1). Here, *k* determines the growth rate of severity: the higher *k* is, the faster the severity value grows as the type of harm becomes more severe. *A* represents the “starting point” of severity: the higher *A* is, the larger the severity value is for the least severe type of harm.

**Table 2:** Exponential regression results of each country’s data. Here, *p* < 0.001 for all parameters. See T1 in S1 File for the full set of regression results.

Country	*A*	*k*	*R* ^2^
Brazil (BR)	40878.84	0.023	0.944
Egypt (EG)	39838.25	0.024	0.909
India (IN)	72922.22	0.015	0.918
Indonesia (ID)	43873.91	0.025	0.894
The Philippines (PH)	37959.63	0.028	0.931
Turkey (TR)	26118.68	0.03	0.95
United States (US)	28545.27	0.029	0.949
Vietnam (VN)	27099.36	0.031	0.914

As shown in Table 2, the regression analysis found that all parameters were statistically significant, thereby confirming the exponential of severity growth across countries. In our sample, Turkey had the highest rate of growth but the lowest “starting point” of severity, while India had the lowest growth rate but the highest “starting point.” The consistent exponential growth means that, contrary to what a simple linear rank order would show, the “distances” between different types of harmful content were not equal—the “distance” between what was ranked in the first place (most severe) and the second place (second most severe) was much larger than that between the 65th place and the 66th place. While a rank order would imply a steady linear growth as a result of assigning consecutive integers, the exponential growth revealed that as the rank grew higher, not only did participants find the behavior more severe, it also gained severity more quickly.

**Fig 2:**
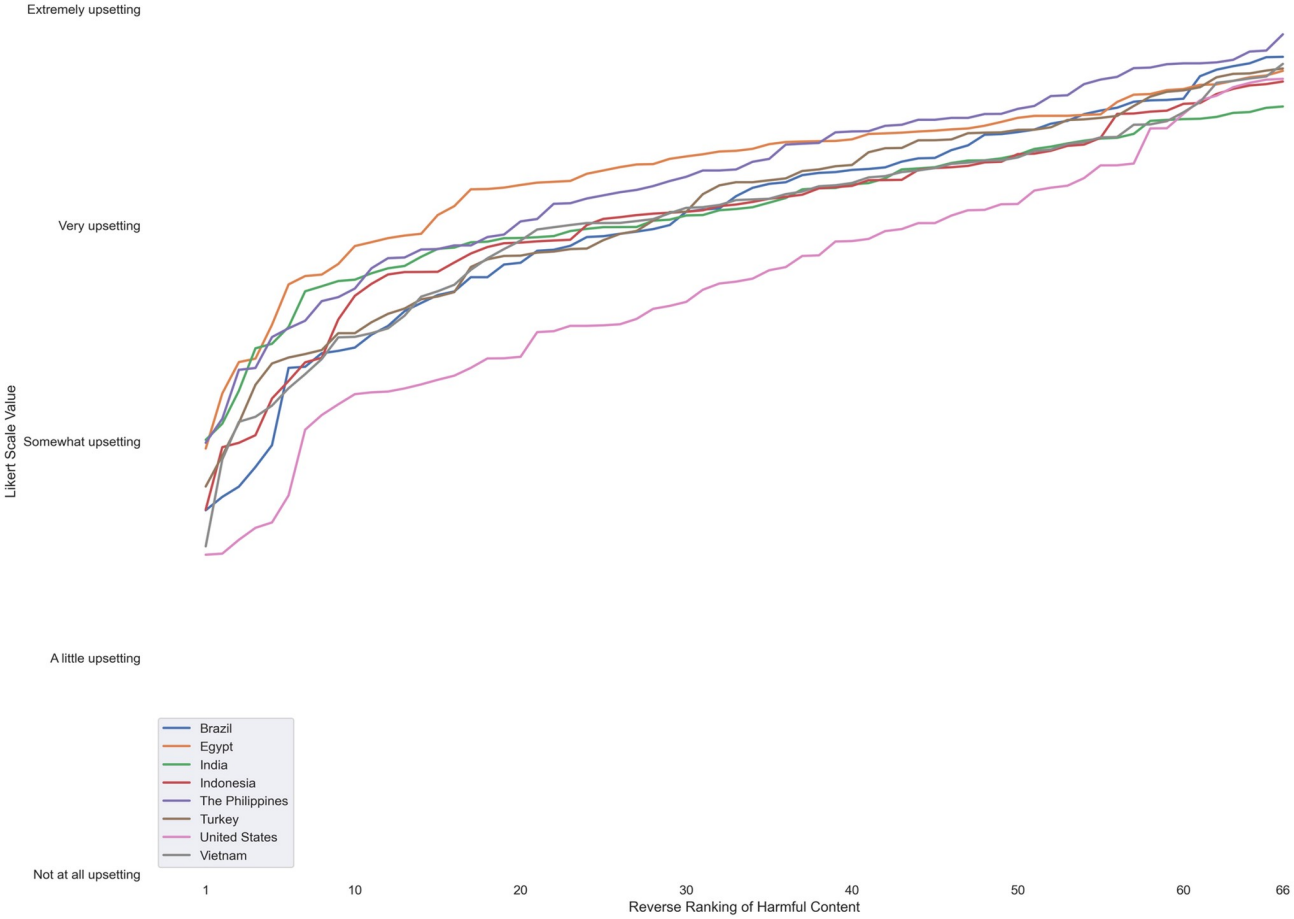
Plot of severity value vs. reverse severity rank order for each country under a Likert-scale measurement.

We also tested the traditional Likert scale measurement as a comparison to the punishment and urgency free-response measurements, and we observed distinctly different relationships. Fig 2 shows a plot of the mean response of each type of harmful content versus its reverse rank order by country; the only difference with Fig 1 is the *y*-axis represents the Likert scale measurement, rather than the free-response measurement. As Fig 2 indicates, while the growth rates of severity still seemed consistent across countries, they were clearly closer to linear growth instead of exponential growth. A possible reason for the distinctive difference in growth rates is the inherent upper limit of Likert scales—in this study, the most severe possible option participants could select was “extremely upsetting,” thereby disallowing free growth. By limiting the higher end of severity, the Likert scale may also flatten the nuanced relationships between different types of harmful content, which the free-response measurements were able to reveal. For example, under the Likert scale, the most severe harm in the U.S. was Child Exploitation Imagery (= 4.68), whose severity value was only 1.88x that of the least severe harm, Engagement Abuse (= 2.48). However, the free-response measurement revealed that, without the arbitrary limit of the higher end, the severity of Child Exploitation Imagery (= 258632.86) was almost 15x that of Engagement Abuse (= 17453.15).

Overall, across the countries we examined, people’s perceptions of harmful content’s severity showed a distinctive pattern under a free-text numeric measurement: when the category of harm was ranked higher, the perceived severity of the harm also grew exponentially. Furthermore, the commonly-used Likert scale measurement concealed the exponential growth by showing a linear growth instead, which would have underestimated the severity of many types of harmful content, especially those on the higher end of severity.

Despite the consistent exponential growth of severity, it is important to note that the specific harmful content in any rank position varied from country to country. In other words, participants in each country had their own ideas of how to rank different harmful content—what was the “worst” varied from country to country. We discuss these differences and similarities between countries in the following sections.

## What are the similarities and differences between countries?

Overall, no two country rankings were the same. There was a lot more disagreement than agreement between countries, and there was always something that any two countries (strongly) disagreed on. However, we were able to observe clusters of countries within which individual countries were more likely to agree with each other.

To identify clusters of countries that are similar, we first calculated each country’s ranking of harmful content, which is determined by the rank order of the average severity value *s*_*k*_ of each type of harmful content *k* across all participants in that country. For example, the most highly ranked harmful content in Vietnam would have the highest average severity value across all Vietnamese participants compared to the other types of harm. See T3 in S1 File for a complete list of country rankings.

**Fig 3:**
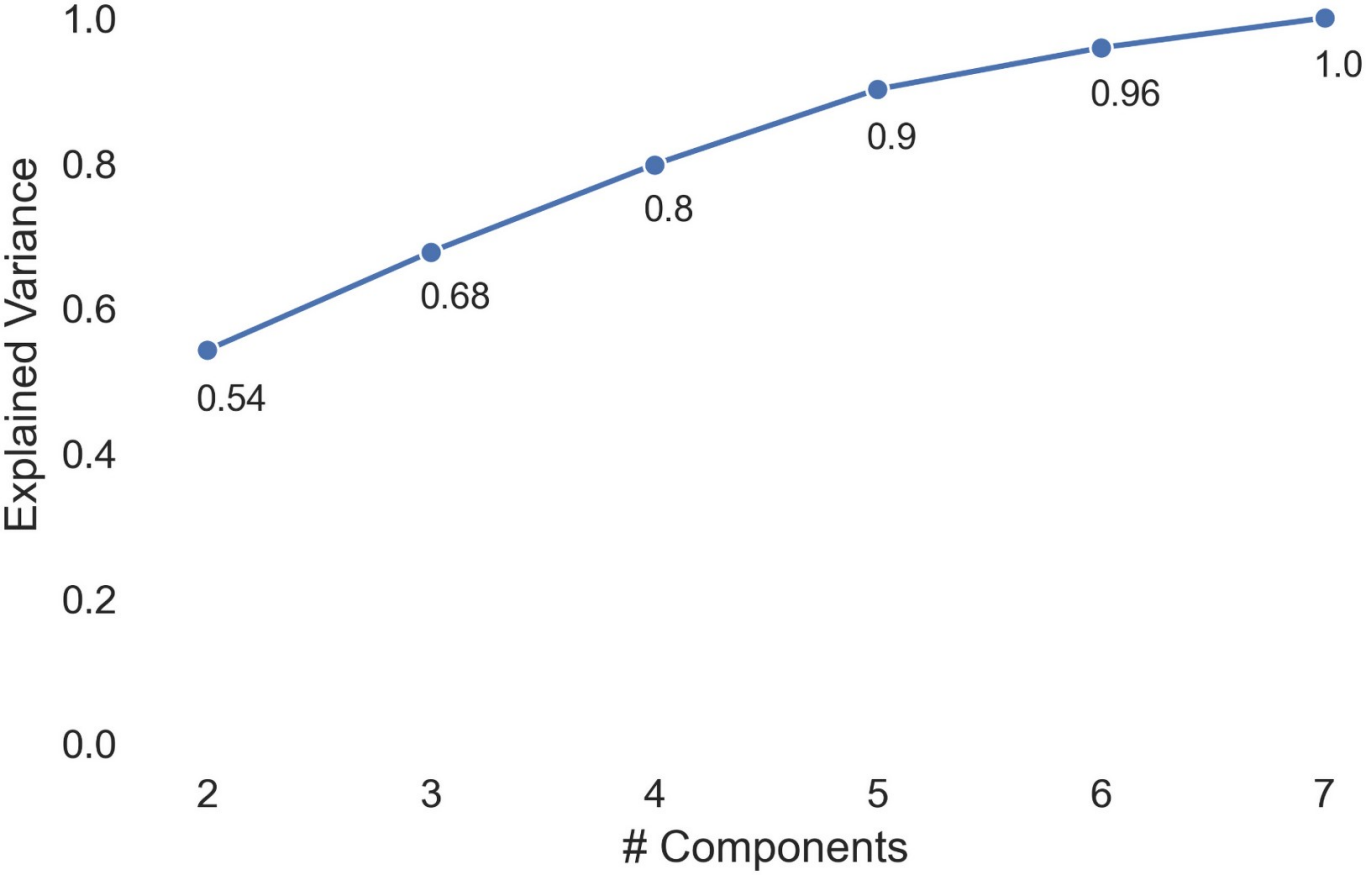
Plot of explained variance vs. number of principal components in PCA.

We then performed principal component analysis (PCA) on these rankings of harmful content by country. PCA is a common technique to reduce dimensionality in a dataset [32], and was necessary because there were far more dimensions (i.e., the number of harmful content types) than data points (i.e., the number of countries), which would make for more expensive computation and less meaningful clusters [33]. We experimented with the number of components *n* ∈ [2, 7] and found that when we projected the original 66-dimensional data onto *n* = 7 components, the result could explain full original variance (shown in Fig 3).

After reducing the number of dimensions from 66 to 7 using PCA, we then used the Gaussian Mixture model to find clusters. We determined the number of clusters *K* by examining the average silhouette coefficient *s* for each possible *K*, which represents how well samples are clustered with samples that are similar to themselves [34]. The higher the silhouette coefficient *s*, the better the clustering is.

**Fig 4:**
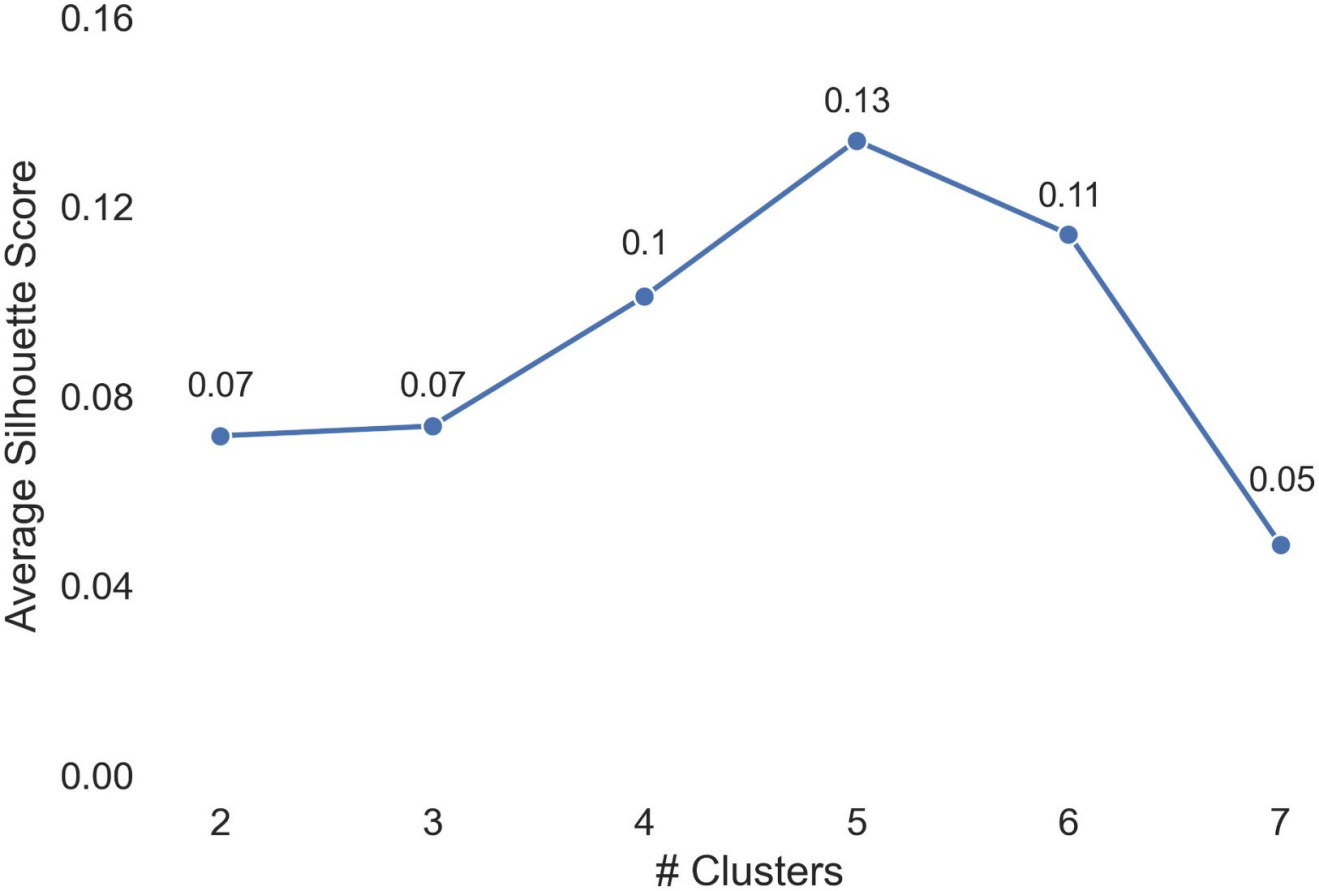
Plot of average silhouette score vs. number of clusters in Gaussian Mixture models.

We tested the Gaussian Mixture models by experimenting with *K* ∈ [2, 7], and found that the best clustering appeared at *K* = 5 (*s* = .13), before the silhouette coefficient started decreasing (shown in Fig 4).

To confirm the robustness of our clustering, we also conducted a pairwise ranking correlation analysis; Table 3 shows the correlation results. The analysis also showed the same clustering, with within-cluster correlations consistently higher than 0.8. In other words, the rankings of countries within each cluster are highly correlated with each other, which corroborates with the Gaussian Mixture clustering result.

**Table 3:** Pairwise ranking correlation results for all countries.

	**BR**	**US**	**IN**	**PH**	**EG**	**VN**	**ID**	**TR**
**BR**	1	0.8835	0.6956	0.8138	0.6641	0.7310	0.6790	0.7660
**US**	0.8764	1	0.7491	0.8421	0.7407	0.6503	0.7017	0.7623
**IN**	0.6956	0.7491	1	0.891	0.8215	0.7606	0.7652	0.7609
**PH**	0.8138	0.8421	0.8910	1	0.8094	0.7586	0.8067	0.7943
**EG**	0.6641	0.7407	0.8215	0.8094	1	0.6336	0.6992	0.6729
**VN**	0.7310	0.6503	0.7606	0.7586	0.6336	1	0.8080	0.7557
**ID**	0.6790	0.7017	0.7652	0.8067	0.6992	0.8080	1	0.7697
**TR**	0.7660	0.7623	0.7609	0.7943	0.6729	0.7557	0.7697	1

Each cluster of countries had its own set of harmful content that it perceived as more or less severe compared to other clusters, based on how they ranked these types of harm. Table 4 lists the types of harmful content perceived as more or less severe by each cluster.

**Table 4:** Country clusters and their ranking characteristics.

Cluster	Harmful Content Perceived as More Severe	Harmful Content Perceived as Less Severe
**Cluster 1** Brazil (BR), United States (US)	Non-consensual Sexual Touching, Graphic Violence: Mutilated Humans, Hate Speech: Exclusion	Adult Non-consensual Intimate Imagery, Marijuana Sale, Drug Use, Vandalism
**Cluster 2** India (IN), The Philippines (PH)	Distributing Virus, Sadism, Drug Use	Suicide Promotion, Creep Shots, Hate Org Coordination
**Cluster 3** Egypt (EG)	Adult Non-consensual Intimate Imagery, Sexual Solicitation, Sexually Explicit Language, Coordinating Harm, Creep Shots, Criminal Group Coordination	Firearm Sale, Graphic Violence: Mutilated Humans, Self-injury Depiction, Harassment
**Cluster 4** Vietnam (VN), Indonesia (ID)	Hate Speech: Dehumanization, False News, Marijuana Sale	Child Nudity, Minor Sexualization, Sadism
**Cluster 5** Turkey (TR)	Eating Disorder Promotion, Eating Disorder Depiction, Harassment, Commercial Spam	Suicide Depiction, Criminal Group Coordination, Criminal Group Propaganda

Overall, we found that each country cluster had a unique set of harmful content that they collectively perceived as more severe or less severe, and these sets rarely overlap between clusters. There are also types of harm that are ranked highly by one cluster, but low in another (e.g., “marijuana sale” has a high ranking in Cluster 4 but a low ranking in Cluster 1).

## On what harmful content do countries agree and disagree?

While we have examined the patterns and differences between countries in terms of how they perceive severity,broadly, it is also valuable to conduct a similar investigation on the rankings of severity for different types of harm. Insights into the agreements and disagreements between individual types of content will reveal which types of harm may deserve more attention and nuanced treatment than others.

**Fig 5:**
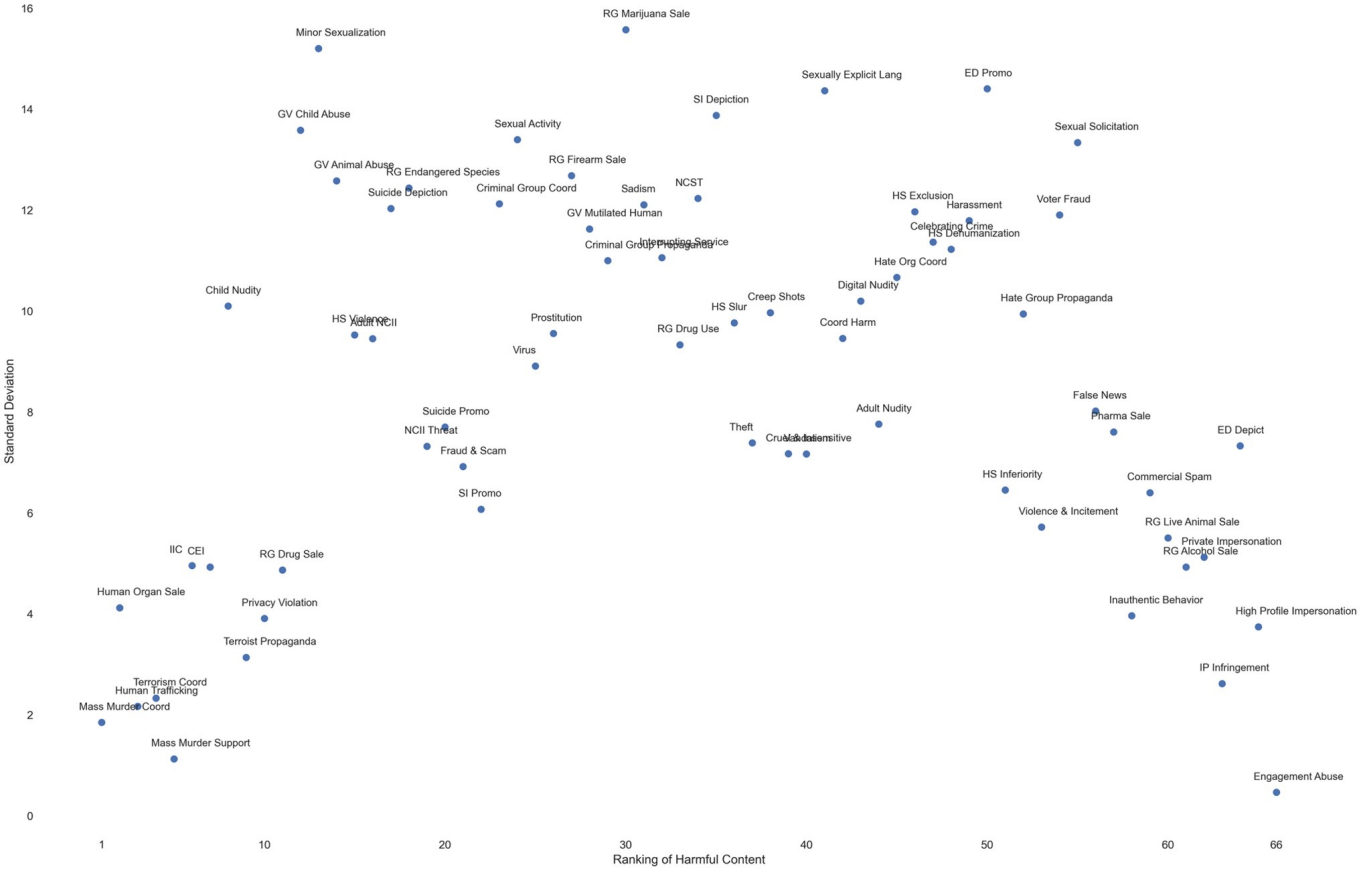
Harmful content’s standard deviation vs. sample-wide ranking.

To examine agreements and disagreements, for each type of harmful content, we calculated standard deviations of all countries’ rankings of that type of content, and plotted it against that type of content’s overall sample-wide ranking (1 = highest), shown in Fig 5. Note that the overall sample-wide ranking here is a country-agnostic one, rather than the aggregate of by-country rankings. The reverse-U shaped curve in Fig 5 shows that countries agreed more on the most severe and the least severe harm, but disagreed more toward the middle (with “marijuana sale” having the largest standard deviation (*SD* = 15.57), and “engagement abuse” (which often appears as clickbaits) having the smallest standard deviation (*SD* = 0.46)).

While standard deviations can give us an general idea of how “spread out” countries are in their rankings of content types, we also examined the maximum extent that countries disagreed on a certain type of content by looking at “outlier” countries that ranked it most extremely. While it is common practice to exclude such outliers in data analysis, we chose to focus on them here because our unit of analysis is an entire country rather than an individual participant. Taking a closer look at the “outliers” will reveal valuable insights into the diversity of perceptions of harmful content. Therefore, we also measured the *max ranking differences*. Here, we define the max ranking difference (Δ*rank*) within a type of content as its lowest ranking (i.e., highest in number) across countries less the highest ranking (i.e., lowest in number) of that content.

**Fig 6:**
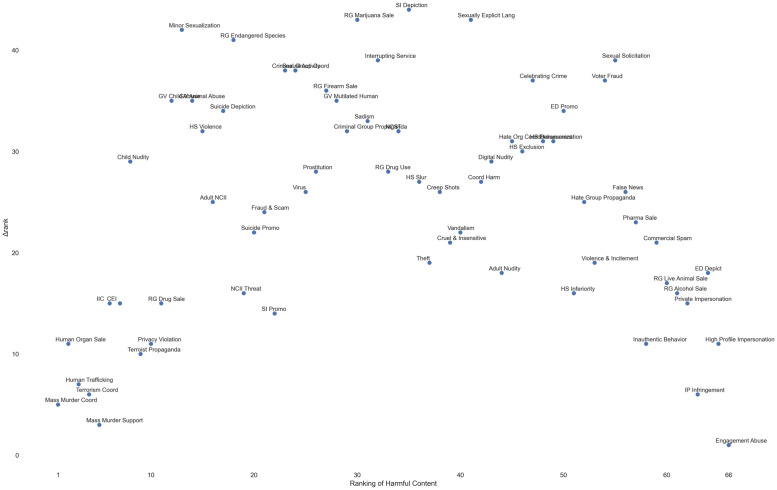
Harmful content’s Δ*rank* vs. sample-wide ranking.

Our analysis showed that the largest Δ*rank* existed in “minor sexualization” and “self injury depiction,” both with Δ*rank* = 45. Similar to our standard deviation analysis, the smallest Δ*rank* appeared in “engagement abuse,” with Δ*rank* = 1. Similar to Figs 5 and 6 shows all 66 types of content’s Δ*rank* plotted against their overall sample-wide severity ranking (1 = highest).

To take a closer look at larger disagreements in Δ*rank*, we counted 18 types of content whose Δ*rank* are at least 33, or half of the total number of rank positions, as shown in Table 5.

**Table 5:** Types of harmful content that had max ranking differences (Δ*rank*) of at least 33, or half of the total number of rank positions.

Harmful Content	Δ*rank*
Minor Sexualization	45
Self Injury Depiction	45
Adult Sexual Activity	43
Regulated Goods: Marijuana Sale	43
Sexually Explicit Language	43
Regulated Goods: Endangered Species Sale	41
Graphic Violence: Mutilated Humans	39
Interrupting Platform Services	39
Voter Fraud	39
Sexual Solicitation	39
Criminal Group Coordination	38
Criminal Group Propaganda	38
Eating Disorder Promotion	38
Celebrating Crime	37
Graphic Violence: Animal Abuse	36
Regulated Goods: Firearm Sale	36
Graphic Violence: Child Abuse	35
Suicide Depiction	34

While there is not a clear pattern in the kinds of content listed in Table 5, it is still worth noting that at least two countries would disagree on whether 19 types of harm, or nearly 30% of all identified types of harm, should be in the top or bottom half.

## What are some country-specific sensitive topics?

The country-level and the content-level analyses in the previous two sections revealed that large disagreements in the perception of severity for different content. While there were clear clusters in perceptions, we found fewer patterns when examining individual types of content. Therefore, in an effort to further seek out patterns in the kinds of content that countries agreed or disagreed on, we employed a content analysis to qualitatively categorize individual types of content into higher-level topic categories.

First, the first two authors each independently categorized all 66 types of harmful content, with an eye toward achieving a reasonably small number of categories to more easily identify patterns. We also tried to follow how “common sense,” rather than moderation experts, might interpret content types. For example, while the Facebook Community Standards [3] categorized “human organ sale” as part of “regulated goods,” an average person may associate it with the black market or mass murder, and in our categorization we followed the latter rationale and categorized it under “Mass Scale Harm.” We then came together to discuss and adjudicate differences, while iterating on the categories. While we acknowledge that our primarily U.S.-centered perspective is likely to have had an influence on our categorizations, we believe it is still a promising first step towards a preliminary understanding of the similarities and differences of individual content rankings in a multi-country context. We eventually agreed upon the following topic categories (see T2 in S1 File for a complete list of harmful content and their topic categories):

- Mass Scale Harm (e.g., Terrorism, Human Organ Sale)
- Vulnerable Groups (e.g., Child Exploitation Imagery, Child Abuse)
- Violence (e.g., Graphic Violence: Mutilated Humans, Sadism)
- Platform Abuse & Spam (e.g., Interrupting Platform Services, Engagement Abuse)
- Sexual Content (e.g., Sexual Activity, Sexually Explicit Language)
- Regulated Goods (e.g., Marijuana Sale, Drug Sale)
- Self-harm (e.g., Suicide Depiction, Eating Disorder Promotion)
- Financial harm (e.g., Fraud & Scam, Privacy Violation)
- Other Directed Harm (e.g., Theft, Vandalism)

Table 6 shows countries’ median ranking of each topic category of content type. Overall, content involving “vulnerable groups” (e.g., children) are consistently the most severe across countries. The perceived high severity is corroborated by the fact that there is at least one type of “vulnerable groups” harm in the top 6 (top 1%) of every country. Interestingly, while “mass scale harm” was only ranked third sample-wide and sometimes even lower in certain countries, it is consistently the majority category of every country’s top 6. A possible reason for the relatively lower ranking of “mass scale harm,” despite its concentration in the most severe harms, is that the category accounts for most *types* of harms (13 out of 66) and is thus more spread out in individual country rankings.

**Table 6:** Countries’ median ranking of each type of harmful content. 1 = most severe; 66 = least severe. The World ranking is a country-agnostic ranking instead of an aggregation from individual country rankings.

Topic Category	Sample	PH	ID	US	IN	BR	VN	EG	TR
Vulnerable Groups	10	9	16	5.5	13	9	18	18.5	9
Financial Harm	15.5	15.5	19.5	15.5	16	19	15	22	17.5
Mass Scale Harm	23	18	25	27	20	19	23	15	42
Self-harm	28.5	33	23.5	30	36.5	31	40.5	31.5	28.5
Violence	31	46	42	23	43	27	40	43	41
Regulated Goods	31.5	35.5	25	42	31	46.5	23	48.5	31.5
Sexual Content	36	32	37.5	32	30.5	39.5	43.5	21.5	37.5
Other Directed Harm	43	41.5	39	46.5	43	38.5	32	43	36
Platform Abuse & Spam	60.5	57.5	56.5	59.5	57.5	59.5	56.5	61.5	61.5

On the other hand, “platform abuse & spam” was consistently perceived as the least severe topic category of harmful content. Specifically, “engagement abuse,” which refers to posting clickbait-like content, was consistently perceived as least severe (except in Vietnam and Egypt, where it was second to last.) Other “platform abuse & spam,” including “intellectual property infringement” and “commercial spam” were also consistently in the bottom 10, showcasing they were not considered severe.

## Discussion and conclusion

Overall, our findings show significant differences in perceptions of harmful content across different countries in the world. The general variability serves as evidence against social media platforms’ current approach of using a single universal set of rules to regulate global users, which falsely implies that people view harmful content consistently across the world. The cross-country disagreement widely existed in different facets of our analysis—in different countries’ rankings of content, in individual types of harmful, and in the higher level topics we used to categorize individual content types.

Despite the general variance, there were some patterns in how users perceived the severity of content. First, regardless of how each country ranked the content types, we found that the perceived severity followed the same rate of growth as the content ranking became increasingly worse—specifically, an exponential growth. Second, while there were no unified ways to describe how all countries ranked harmful content, there were country clusters where the rankings were similar, which suggests the possibility of moderation by country groups. Finally, in our analysis of the agreement and disagreement on the level of individual types of content, as well as high-level topics, the convergence toward the extreme ends suggests that people had more consensus on the most severe and the least severe types of harm, but had less consensus toward the content in the middle.

Taken together, our findings have several implications for platform content moderation. First, the consistently exponential growth across all countries provides a principle for moderation prioritization in localized contexts—while what types of harm should be prioritized may vary from country to country, the underlying relationship between the types of harmful content may remain the same. The distinctive exponential growth also shows that using a simple ranking approach may be misleading because it implies a linear growth of severity and thus highly severe content may not receive the attention that it deserves.

Furthermore, our findings indicate that policymakers of social media platforms, who likely developed community guidelines from a predominantly Western (and particularly U.S.) point of view [1], may deprioritize the moderation of content that is perceived as highly severe in non-U.S. countries. However, we am not claiming that platform policy making should be in a “U.S. vs. elsewhere” fashion, because our evidence shows that perceptions of harmful content are complex and highly varying in countries outside the U.S. The risk of careless categorization is even more pronounced given that moderation resources and capacity are inevitably limited. Therefore, we argue that a promising direction for content moderation is to customize and localize by countries. At the same time, we also acknowledge that differences may still exist within countries, and more research is needed to uncover the specific nuances in any particular country. We also encourage the platform to and critically consider the value of adopting user feedback on content moderation, which we expand on later in this section.

Our analysis of country-specific sensitive topics revealed a similar pattern of consistent opinions about the most severe (“mass scale harm” and “vulnerable groups”) and the least severe (“platform abuse” and certain kinds of “regulated goods”) harm, but diverse opinions about the middle rankings. However, we present these findings as evidence for moderation approaches cautiously. While platforms may want to prioritize harmful content involving “mass scale harm” and “vulnerable groups” (as they may already be doing), it would be perilous to treat the least severe content as “not important,” because our participants, regular social media users, may be unaware of the non-obvious harms that they could cause (e.g., “sale of prescription drugs” or “intellectual property infringement”). Further, the variance of perceptions of severity show the need for further research on understanding diversely perceived harms, as these harms are likely to be more complex. Therefore, simplistic moderation strategies for them may unintentionally privilege certain groups of people at the cost of others, a point echoed by Schoenebeck et al. in their study of different people’s perceptions of justice models [35].

### Methodological implications

On a methodological level, researchers and practitioners may find value in adopting the free-text numeric measurement method that we used within their own platforms or communities. By assigning unbounded values to individual types of harmful content, our method was able to reveal quantified relationships between values (e.g., one case of “child exploitation imagery” is equivalent to *x* cases of “commercial spam” in terms of severity) that the Likert scale could not. While reductive and not able to capture the full nuance of harmful content, these equivalence relationships can be a promising first step in guiding decisions in large-scale content moderation, including moderator resource allocation, proactive detection, and response prioritization, for different kinds of content, especially given the inherent limitation in human moderation capacity [1]. Researchers and practitioners can also conduct the same measurement survey with diverse groups of stakeholders, such as users, moderators, and policy experts, to gain a variety of perspectives. In the context of platform moderation, multi-stakeholder measurement may prove especially fruitful, because regular users who rarely encounter highly severe types of harm may not be able to reasonably estimate their impacts.

### Ethical implications

Our study also has important ethical implications. While we have identified *what* types of behavior were sensitive and important for specific countries, we want to emphasize that we present no evidence for *why* they are important, which should be seen as a limitation of our study. We also intentionally avoided speculating possible reasons for the severity rankings we found. Irani et al. argued for a generative, rather than taxonomic, view of culture that positions people at the intersection of cultures [36]. Even though we conducted the study with some inherent regional taxonomies, geographically co-located individuals still experience and produce different, dynamic, and sometimes overlapping cultures. Therefore, reckless assumptions and speculations about “cultures” may not only be incorrect, but also highly dangerous, because they may impose harmful stereotypes and stigma on people. In the case of content moderation, the stakes are even higher because the resulting stigma may be associated with highly severe, possibly illegal behavior. Therefore, we caution against simplistic interpretations of these regionally different topics, such as assigning cultural stereotypes to these differences (e.g., “Vietnam users perceive ‘creep shots’ less severely than other countries because of their certain cultural values”). we urge researchers to conduct careful, deep-dive case studies into particular types of behavior to understand their full complexity.

Nevertheless, our research provides insights into the views of international users in platform policymaking, which has otherwise been centered around compliance with various laws and regulations. While it is unclear how exactly platform-level moderation policies were made, or whether user inputs were taken into account in the making of these policies, given that platforms currently do not have customized policies for different countries, it is reasonable to suspect that U.S. perspectives are weighed more heavily than those from other parts of the world, given the majority of large social media companies are headquartered in the U.S. However, the non-overlap and the opposite ranking of certain harms in our clustering analysis showed the necessity of treating different countries differently in creating rules and policies, rather than taking a one-size-fits-all approach that is likely to deprioritize harmful content that certain countries perceive as highly severe. Similarly, both our standard deviation analysis and the Δ*rank* analysis showed the necessity of customizing content moderation by country. A universal approach is likely to mistreat a nontrivial amount of harmful content by dismissing cross-country differences, and the level of harm across content types on which people heavily disagreed deserves greater attention, more in-depth research, and more nuanced treatment. Overall, our research complicates and challenges the current practice of blanket content policies for the whole world by revealing the diverse attitudes toward harmful content from users from different countries.

At the same time, it is important to note that universal approaches can sometimes be ideal, as over-customization may result in fragmented and inefficient content regulation policies. Our results also showed that certain issues such as child exploitation and the killing of humans indeed received cross-country consensus, which indicate blanket moderation may be advisable for similar issues. While there are no clear answers, platforms should carefully consider the trade-off between uniformity and customization, while keeping in mind the specific abuse they are dealing with, as well as current thorny debates such as online safety vs. freedom of speech.

The trade-off between universal and relativist content moderation policies and related ethical implications will be an everlasting one. While we have argued that regional differences are important considerations, it is also crucial to not assume that localized norms or laws are necessarily ethical. Our method does not speak to how to *resolve* conflicts between regional norms—rather, it helps *surface* and *highlight* these conflicts. Our method can be a way for platforms to understand how people in different countries quantify harmful or objectionable content, but in the end, platforms need to decide how best to reconcile regional differences, what norms align with platform values and priorities, and what they consider “right.” For example, death sentences for LGBTQ people may be acceptable in some locations or cultures [37], but that does not justify acts of genocide. However, it is certainly unacceptable in many other cultures, including in the U.S. where many tech companies are based [38]. Further, platforms should not take rankings of harmful content at face value, because a value being accepted does not make it right, and platforms should strive for more nuanced understandings beneath any stated value.

It is critical to note that neither the approach nor the findings in our study should be used as the sole reason to determine whether or not a piece of content should be moderated. Nor the findings alone determine the degree of harshness to which one should be punished for posting harmful content. we raise these precautions for two reasons: First, the question that why people perceived the harmful content as such remains unanswered. Second, more importantly, the perceptions of regular internet users should not be the gold standard of policymaking or the right way forward.

### Implications for platform content moderation

The complexity of analyzing harmful content globally may be exacerbated by the inability to describe and interpret regionally, reflecting Fricker’s [39] notion of hermeneutic injustice. Fricker defines hermeneutic injustice as when someone “has a significant area of their social experience obscured from understanding owing to prejudicial flaws in shared resources for social interpretation.” In other words, a group of people may not have language or ability to describe and interpret a nuanced social experience that they do regularly experience. Fricker uses the example of stalking to demonstrate the concept of hermeneutic injustice. A man does not perceive stalking of a woman as harmful because the woman cannot describe her experience in the man’s own interpretive system, and the man who never experienced stalking may have no idea what harm stalking actually entails. The same hermeneutic injustice is likely to apply in the context of content moderation as well. For example, people who are not aware of the consequences of anorexia nervosa may perceive its depiction as simply someone being “skinny” and therefore do not see it as being harmful.

Hermeneutic injustice also has broader consequences, because much of the platform policymaking relies on translation rather than local development. While platform policies do exist in multiple languages, as opposed to being developed organically by people across the world who are familiar with local contexts, they are often translated from English with a U.S. perspective, and essentially become different versions of U.S. ideals—not to mention how much information might be lost in the translation. The survey in our study suffers from the same epistemic limitation: It asked users to rate scenarios containing harmful content, but these scenarios, as well as the harmful content categories from which these scenarios arise, are often conceptualized and described by social media platforms in a small part of the world (in this case, the urban United States). People in the U.S. may perceive these categories and scenarios as comprehensive and representative, but it is possible that they do not even start to describe the harm and abuse that people in other parts of the world experience—it may not even be possible to describe them in English. It is also likely that people in other parts of the world cannot understand the harm that the U.S.-centric policies describe because they do not exist in their interpretive systems. Therefore, while our findings reveal that perceptions of harmful content can be different across countries, they should not be the predominant factor in policymaking. Content moderation practices cannot fully protect people if they are only imparting a specific idealized version of the harms these people experience. Policies for any country should be developed with local perspectives and in local terms.

Beyond hermeneutic injustice, sometimes users’ values may simply go against the values that a platform or society holds, in which case it is in the platform’s best interest to not take these user inputs into account in making platform policies. While research regarding social media platforms has historically valued soliciting user inputs and fulfilling them, there is no guarantee that user perceptions are always going to be beneficial (or even legal), especially in determining what content is welcome or unwelcome on a platform. More likely than not, platforms will not want to implement perspectives from users who want to see child pornography, who support ethnic cleansing, or who think anything that does not pertain to a certain religious belief should be removed. While these are extreme examples, some of the surprising findings in our study, such as the depiction of “eating disorders” being lowly-rated in the United States, indicate that it is possible for some users to hold values that platforms may determine as harmful by value or by law. While these values may become less apparent when they are aggregated into large amounts of user inputs, but still have the potential to deprioritize harms that the platform may want to be prioritized.

## Supporting information

S1 File Additional analysis details. (PDF)

S2 File Full survey instrument and participant response data. (ZIP)
