# Supplementary material for: Understanding international perceptions of the severity of harmful content online
Source: PLoS One. 2021 Aug 27;16(8):e0256762. doi: 10.1371/journal.pone.0256762 (PMC8396792; doi:10.1371/journal.pone.0256762)
Supplement: S1 File — (PDF) [file pone.0256762.s001.pdf]

T1. Results from the exponential regressions. Note that in order to fit $y = Ae^{kx}$, we transformed the equation to a linear regression form by taking the natural logarithm on both sides ($ln\, y = ln\, A + kx$). As such, $A = e^{const}$.This table only provides the results of the linear regression form.

Region	Parameter	Coef	Std Err	t	P>|t|	[0.025	0.975]	R^2
Brazil	const	10.618	0.027	392.257	0.000	10.564	10.672	0.944
	k	0.023	0.001	33.235	0.000	0.022	0.025	
Egypt	const	10.593	0.036	295.543	0.000	10.521	10.664	0.909
	k	0.024	0.001	26.463	0.000	0.023	0.026	
India	const	11.197	0.022	517.915	0.000	11.154	11.240	0.918
	k	0.015	0.001	27.611	0.000	0.014	0.017	
Indonesia	const	10.689	0.040	269.090	0.000	10.610	10.768	0.894
	k	0.025	0.001	24.024	0.000	0.023	0.027	
The Philippines	const	10.544	0.038	278.222	0.000	10.469	10.620	0.931
	k	0.028	0.001	29.519	0.000	0.027	0.031	
Turkey	const	10.170	0.033	312.823	0.000	10.105	10.235	0.950
	k	0.030	0.001	36.765	0.000	0.029	0.033	
United States	const	10.259	0.033	307.036	0.000	10.192	10.326	0.949
	k	0.029	0.001	34.122	0.000	0.028	0.031	
Vietnam	const	10.207	0.045	224.853	0.000	10.117	10.298	0.914
	k	0.031	0.001	26.693	0.000	0.029	0.034	

T2. Complete list of topic categories and the types of harmful content under each category.

Topic Category	Count	Percentage of all 66 content types	Harmful Content
Directed Harm	8	0.12	Cruel and Insensitive
			Harassment
			Hate Speech: Dehumanization
			Hate Speech: Exclusion
			Hate Speech: Inferiority
			Hate Speech: Slur
			Theft
			Vandalism
Financial Harm	2	0.03	Fraud and Scam
			Privacy Violation
Mass Scale Harm	13	0.20	Coordinating Harm
			Criminal Group Coordination
			Criminal Group Propaganda
			False News
			Hate Group Propaganda
			Hate Org Coordination
			Human Trafficking
			Mass Murder Coordination
			Mass Murder Support
			Reg Goods: Human Organ Sale
			Terrorism Coordination
			Terrorist Propaganda
			Voter Fraud
Platform Abuse & Spam	8	0.12	Commercial Spam
			Disseminating Virus
			Engagement Abuse
			High Profile Impersonation
			Inauthentic Behavior
			Interrupting Platform Service
			IP Infringement
			Private Impersonation

Regulated Goods	8	0.12	Pharma Sale
			Reg Goods: Alcohol Sale
			Reg Goods: Drug Sale
			Reg Goods: Endangered Species Sale
			Reg Goods: Firearm Sale
			Reg Goods: Live Animal Sale
			Reg Goods: Marijuana Sale
			Regulated Goods: Drug Use
Self-harm	6	0.09	Eating Disorder Depiction
			Eating Disorder Promotion
			Self-injury Depiction
			Self-injury Promotion
			Suicide Depiction
			Suicide Promotion
Sexual Content	10	0.15	Adult Non-Consensual Intimate Imagery
			Adult Nudity
			Creep Shots
			Digital Nudity
			NCII threat
			Non-Consensual Sexual Touching
			Prostitution
			Sexual Activity
			Sexual Solicitation
			Sexually Explicit Language
Violence	5	0.08	Celebrating Own Crime
			Graphic Violence: Mutilated Humans
			Hate Speech: Violence
			Sadism
			Violence and Incitement

Vulnerable Groups	6	0.09	Child Exploitation Imagery
			Child Nudity
			Graphic Violence: Animal Abuse
			Graphic Violence: Child Abuse
			Inappropriate Interactions with Children
			Minor Sexualization

T3. Sample-wide and each region's ranking of harmful content. Note that the sample-wide ranking is a region-agnostic one (i.e., ranking of mean severity value regardless of region), rather than the aggregation of regional rankings.

Harmful Content	Sample	Brazil	Egypt	India	Indonesia	The Philippines	Turkey	United States	Vietnam
Mass Murder Coord	1	3	6	1	3	1	5	5	3
Human Organ Sale	2	4	1	2	2	2	9	12	1
Human Trafficking	3	5	4	4	9	7	4	4	2
Terrorism Coord	4	7	3	3	1	6	7	7	6
Mass Murder Support	5	6	5	5	6	3	6	6	4
IIC	6	1	2	6	4	5	1	2	16
CEI	7	2	16	10	8	8	3	1	8
Child Nudity	8	8	17	31	14	4	2	3	20
Terrorist Propaganda	9	9	9	12	5	13	8	15	10
Privacy Violation	10	14	11	16	7	9	17	13	18
RG Drug Sale	11	20	8	9	11	11	10	17	5
GV Child Abuse	12	13	44	14	39	10	20	9	13
Minor Sexualization	13	10	20	50	12	12	15	8	38
GV Animal Abuse	14	12	45	18	32	14	16	10	33
HS Violence	15	15	14	8	40	19	19	22	25
Adult NCII	16	32	7	30	10	23	12	26	22
Suicide Depiction	17	11	25	21	22	15	45	11	36
RG Endangered Species	18	17	48	26	35	20	32	24	7
NCII Threat	19	16	13	27	13	29	13	29	19
Suicide Promo	20	22	28	29	34	30	30	16	12

Fraud & Scam	21	24	35	22	25	22	18	18	11
SI Promo	22	25	18	15	18	28	14	28	14
Criminal Group Coord	23	19	15	24	20	18	53	27	31
Sexual Activity	24	40	10	7	21	16	24	21	45
Virus	25	34	26	41	15	17	21	20	21
Prostitution	26	41	21	13	17	24	23	35	17
RG Firearm Sale	27	21	51	25	16	39	25	38	15
GV Mutilated Human	28	27	49	40	43	48	34	14	37
Criminal Group Propaganda	29	30	23	34	27	27	55	43	24
RG Marijuana Sale	30	45	40	23	24	32	11	52	9
Sadism	31	29	24	52	19	21	43	23	40
Interrupting Service	32	56	31	17	26	34	36	31	35
RG Drug Use	33	48	37	20	28	26	31	44	32
NCST	34	18	33	38	41	38	50	19	50
SI Depiction	35	44	55	11	38	36	22	42	44
HS Slur	36	35	46	19	44	45	26	37	30
Theft	37	47	39	28	29	33	35	45	29
Creep Shots	38	39	22	45	48	44	29	25	42
Cruel & Insensitive	39	28	38	49	46	31	37	41	34
Vandalism	40	43	30	35	33	40	39	48	26
Sexually Explicit Lang	41	51	12	42	30	25	46	36	55
Coord Harm	42	46	19	36	23	37	42	39	41
Digital Nudity	43	38	29	39	50	47	56	47	27
Adult Nudity	44	49	32	37	31	35	49	46	47
Hate Org Coord	45	36	36	33	52	61	44	30	48
HS Exclusion	46	26	56	44	56	51	33	33	54

Celebrating Crime	47	23	43	54	60	46	40	54	43
HS Dehumanization	48	54	41	32	51	52	38	59	28
Harassment	49	33	59	58	36	42	28	51	52
ED Promo	50	37	34	60	61	55	27	32	59
HS Inferiority	51	42	53	47	42	41	48	56	57
Hate Group Propaganda	52	31	42	56	55	54	54	34	46
Violence & Incitement	53	53	47	43	62	50	51	53	56
Voter Fraud	54	52	54	46	45	60	59	55	23
Sexual Solicitation	55	64	27	51	37	43	57	49	66
False News	56	50	58	48	59	49	52	65	39
Pharma Sale	57	57	52	61	47	53	58	40	63
Inauthentic Behavior	58	55	61	53	58	59	60	62	51
Commercial Spam	59	58	60	55	57	56	41	58	62
RG Live Animal Sale	60	59	66	63	54	62	62	57	49
RG Alcohol Sale	61	65	57	62	49	63	61	60	58
Private Impersonation	62	61	64	59	53	58	65	50	60
IP Infringement	63	63	63	57	63	57	63	61	61
ED Depict	64	60	50	65	65	65	47	64	64
High Profile Impersonation	65	62	62	64	64	64	64	63	53
Engagement Abuse	66	66	65	66	66	66	66	66	65
