# Supplementary material for: Understanding international perceptions of the severity of harmful content online
Source: PLoS One. 2021 Aug 27;16(8):e0256762. doi: 10.1371/journal.pone.0256762 (PMC8396792; doi:10.1371/journal.pone.0256762)
Supplement: S2 File — (ZIP) [file pone.0256762.s002.zip › Survey Instrument.pdf]

Commitment Screener

We care about the quality of our data, in order for us to get the most accurate measures of your opinions, it is important that you thoughtfully provide your best answers to each question in this survey.

Do you commit to thoughtfully provide your best answers to each question in this survey?

- I will provide my best answers
- I will NOT provide my best answers
- I can't promise either way

Social Media Use Screener

Which of the following social media platforms you have used in the last 30 days? Please select all that apply.

- Twitter
- Snapchat
- Reddit
- Facebook
- Instagram
- YouTube
- WhatsApp
- None of the above

Instruction

Welcome and thank you for agreeing to participate in this research study. Your participation is important to us, and we are thankful for your time and effort.

Please read carefully.

Throughout this survey, we will show you examples of different kinds of online content. Along with each example there will be three questions to answer about that content. The examples have been formatted to look like posts from one of social media platforms you have used in the last 30 days: **Facebook.**

For the first question, you will need to decide how much money you would fine the person who posted the content as a punishment. The amount of money fined should reflect the penalty you feel this person deserves for posting the content. **In other words, the worse you feel the content is, the more you should fine the person.**

For the second question, you will need to decide how much money Facebook should spend to make sure the content gets removed immediately over other types of content. The amount of money should reflect the urgency of reviewing and potentially removing that content. In other words, **the worse you feel the content is, the more Facebook should spend to prioritize it.**

For the third question, you will need to answer how upsetting the content is to you personally.

For the first two questions, **there is no limit to the amount you may decide. Zero is also an acceptable answer** if you don't think the content is bad or violates any Facebook rules.

As a reminder, your participation is voluntary. Your responses are anonymous and you may choose to stop participating at any time.

Before we begin the main survey, we would like you to complete some practice examples. In the practice as well as the main survey, the content examples will vary, but the questions to answer will always be the same. The button below will take you to the first practice question.

Scenario Questions

For the practice questions and the main survey, participants will answer the following three questions for every scenario presented. Note that "{{local currency}}" will be replaced with the currency of the participant's country. For example, U.S.-based participants will see "U.S. Dollars (USD)," and Vietnam-based participants will see "đồng Việt Nam (VND)."

- How much money, if any, would you fine the person who posted this content? Please indicate your answer in {{local currency}}. You only need to enter a number. (Free-text response)
- How much money, if any, do you think Facebook should spend to remove this content immediately over other types of content? Please indicate your answer in {{local currency}}. You only need to enter a number. (Free-text response)
- How upsetting is this content to you, if at all?
    - Extremely upsetting
    - Very upsetting
    - Somewhat upsetting
    - A little upsetting
    - Not at all upsetting

Practice Scenarios

Every participant will see the following two practice scenarios, and answer the three questions in Scenario Questions for each scenario.

1. Imagine you saw: a wedding photo on Facebook
2. Imagine you saw: a post of congratulations on a friend's job announcement on Facebook.

For these two scenarios, if the participant gives a non-zero answer to the first question (fine), they will see the following text on the next screen:

You chose to fine the person in the last practice question. Did you think the content was bad or violated Facebook rules? **Remember, feel free to enter 0 if you don't think the content is bad or violates Facebook rules.**

If the participant gives a non-zero answer to the second question (Facebook spending), they will see the following text on the next screen:

You indicated that Facebook should spend money to remove the content immediately in the last practice question. Did you think the content was bad or violated Facebook rules? **Remember, feel free to enter 0 if you don't think the content is bad or violates Facebook rules.**

Main Survey Scenarios

Each participant will randomly see half of the following 66 scenarios, and answer the three questions in Scenario Questions for each scenario.

1. Imagine you saw: **child pornography** on Facebook.
2. Imagine you saw: a photo of **someone displaying pornography to children** on Facebook.
3. Imagine you saw: a photo of **a minor in a sexual pose** on Facebook.
4. Imagine you saw: a post that **encourages people to commit suicide** on Facebook.
5. Imagine you saw: a photo of **someone committing suicide** on Facebook.
6. Imagine you saw: **an invitation to participate in terrorist activities** on Facebook.
7. Imagine you saw: **an invitation to a hate group gathering** on Facebook.
8. Imagine you saw: a post that **attempts to sell children** on Facebook.
9. Imagine you saw: a post of **a plan to kill multiple people** on Facebook.
10. Imagine you saw: a photo of **revenge porn** on Facebook.
11. Imagine you saw: a post that **threatens to show someone's revenge porn** on Facebook.
12. Imagine you saw: a photo of **someone sexually touching a drunk person** on Facebook.
13. Imagine you saw: a post that **celebrates a terrorist group** on Facebook.
14. Imagine you saw: a post that **celebrates the killing of multiple people** on Facebook.
15. Imagine you saw: a post that **celebrates a hate group** on Facebook.
16. Imagine you saw: a post that **calls for help to beat someone up** on Facebook.
17. Imagine you saw: a post that says **people from a certain country should die** on Facebook.
18. Imagine you saw: a post that **brags about hurting someone** on Facebook.
19. Imagine you saw: a post that says **people from a certain country are insects** on Facebook.
20. Imagine you saw: a post that **expresses enjoyment of other people suffering from violence** on Facebook.
21. Imagine you saw: a post that **encourages people to starve themselves** on Facebook.
22. Imagine you saw: a post that **encourages people to cut themselves** on Facebook.
23. Imagine you saw: a photo of **someone starving themselves** on Facebook.
24. Imagine you saw: a photo of **someone cutting themselves** on Facebook.
25. Imagine you saw: a post that **attempts to sell cocaine** on Facebook.
26. Imagine you saw: a post that **attempts to sell prescription drugs** on Facebook.
27. Imagine you saw: a post that **scams people into sending money** on Facebook.

28. Imagine you saw: a post of **false information about who can vote in an election** on Facebook.
29. Imagine you saw: a post that **encourages people to damage public properties** on Facebook.
30. Imagine you saw: a post that **pretends to come from a celebrity** on Facebook.
31. Imagine you saw: a post that **pretends to come from your friend** on Facebook.
32. Imagine you saw: **a secretly taken photo up someone's skirt** on Facebook.
33. Imagine you saw: a post that **harasses someone** on Facebook.
34. Imagine you saw: a post that says **people from a certain country are mentally ill** on Facebook.
35. Imagine you saw: a post that says **people from a certain country should be segregated** on Facebook.
36. Imagine you saw: a post of **racial slurs** on Facebook.
37. Imagine you saw: a post that **makes fun of other people's disabilities** on Facebook.
38. Imagine you saw: a photo of **someone beating children** on Facebook.
39. Imagine you saw: a photo of **a dismembered body on a street** on Facebook.
40. Imagine you saw: a post that **attempts to sell guns** on Facebook.
41. Imagine you saw: a post that **leaks someone's password for their bank account** on Facebook.
42. Imagine you saw: a post that **offers prostitution services** on Facebook.
43. Imagine you saw: a post that **attempts to sell marijuana** on Facebook.
44. Imagine you saw: a post of **a dismembered body in a hospital** on Facebook.
45. Imagine you saw: a photo of **someone torturing an animal** on Facebook.
46. Imagine you saw: a photo of **naked children** on Facebook.
47. Imagine you saw: a post that **asks for sex partners** on Facebook.
48. Imagine you saw: a photo of **adults having sex** on Facebook.
49. Imagine you saw: a post of **detailed description of sexual intercourse** on Facebook.
50. Imagine you saw: posts of **repeated false advertising** on Facebook.
51. Imagine you saw: a post that claims **people have to like something before they can see it** on Facebook.
52. Imagine you saw: a photo of **naked adults** on Facebook.
53. Imagine you saw: a picture of **animated pornography** on Facebook.
54. Imagine you saw: a post of **fake news** on Facebook.
55. Imagine you saw: **a link to download a pirated movie** on Facebook.
56. Imagine you saw: a post that **teaches people how to steal a car** on Facebook.
57. Imagine you saw: an invitation to **participate in a criminal group gathering** on Facebook.
58. Imagine you saw: a post that **celebrates a criminal group** on Facebook.
59. Imagine you saw: a post that **attempts to sell human organs** on Facebook.
60. Imagine you saw: a post that **attempts to sell live animals not from pet stores** on Facebook.
61. Imagine you saw: a post that **attempts to sell endangered animals** on Facebook.
62. Imagine you saw: a post that **encourages people to participate in a highly dangerous activity** on Facebook.

63. Imagine you saw: a post with **a link to a site with a virus** on Facebook.
64. Imagine you saw: a post that **attempts to sell alcohol and cigarettes not from a store** on Facebook.
65. Imagine you saw: a post that **misleads people about the purpose of its content** on Facebook.
66. Imagine you saw: a post that **teaches people how to hack Facebook** on Facebook.

Demographics

Lastly, we would like to know a little more about you.

What is your gender? (Select all that apply)

- Woman
- Man
- Non-binary
- Prefer not to disclose
- Prefer to self-describe: [text box]

What is your age?

- 18-24
- 25-34
- 35-44
- 45-54
- 55-64
- 65-74
- 75-84
- 85 or older

What best describes you? (Select all that apply)

- American Indian or Alaska Native
- Asian
- Black or African American
- Native Hawaiian or Pacific Islander
- White
- Other: [text box]

What is the highest level of education you have completed?

- Less than a high school diploma
- High school diploma or GED
- Some college
- College graduate
- Some postgraduate work
- Postgraduate degree
